# 3-Ethyl-8-meth­oxy-4-(2,3,4,6-tetra-*O*-acetyl-β-d-glucopyranos­yloxy)quinolin-2(1*H*)-one

**DOI:** 10.1107/S1600536810016636

**Published:** 2010-05-12

**Authors:** Roman Kimmel, Marek Nečas, Stanislav Kafka, Janez Košmrlj, Robert Vícha

**Affiliations:** a Department of Chemistry, Faculty of Technology, Tomas Bata University in Zlin, Nám. T. G. Masaryka 275, Zlín,762 72, Czech Republic; b Department of Chemistry, Faculty of Science, Masaryk University in Brno, Kamenice 5, Brno–Bohunice, 625 00, Czech Republic; c Department of Chemistry, Faculty of Chemistry and Chemical Technology, University of Ljubljana, Aškerčeva 5, 1000 Ljubljana, Slovenia

## Abstract

The structure of the title compound, C_26_H_31_NO_12_, contains an essentially planar quinoline skeleton, with the maximum deviation from the best plane being 0.055 (2) Å, and an oxane ring in a classical chair conformation with the following Cremer and Pople puckering parameters: *Q* = 0.586 (2) Å, θ = 11.5 (2)° and ϕ = 309.4 (10)°. One acetyl group displays rotational disorder with occupancies of 0.634 (8):0.366 (8). The crystal packing is stabilized by N—H⋯O hydrogen bonds, which link mol­ecules into chains along the *a* axis. The packing is further stabilized by weak C—H⋯O interactions. The absolute configurations on the carbons in the oxane ring correspond to those of the commercial starting material and are unchanged in the well known mechanism of the Koenigs–Knorr synthesis.

## Related literature

For the synthesis of related compounds and their biological activity, see Kimmel *et al.* (2010 ▶); Suzuki *et al.* (2007 ▶). For puckering parameters, see Cremer & Pople (1975 ▶).
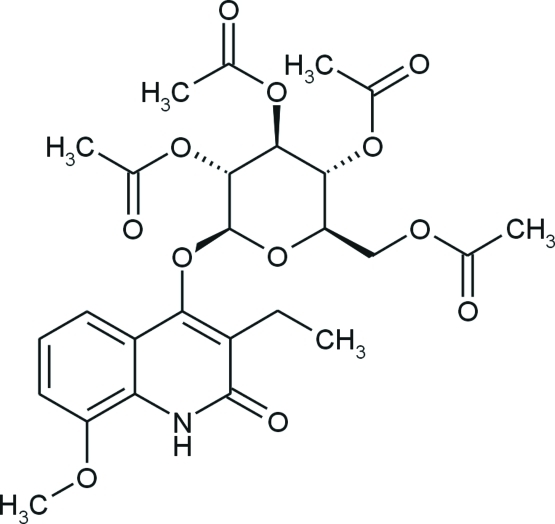


## Experimental

### 

#### Crystal data

C_26_H_31_NO_12_
*M* *_r_* = 549.52
Orthorhombic, 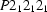

*a* = 5.36993 (11) Å
*b* = 19.2205 (6) Å
*c* = 27.2479 (6) Å
*V* = 2812.33 (11) Å^3^
*Z* = 4
Mo *K*α radiation
μ = 0.10 mm^−1^
*T* = 150 K
0.40 × 0.40 × 0.30 mm

#### Data collection

Kuma KM-4 CCD diffractometer
Absorption correction: multi-scan (*CrysAlis RED*; Oxford Diffraction, 2006 ▶) *T*_min_ = 0.918, *T*_max_ = 0.967
32021 measured reflections
3429 independent reflections
2990 reflections with *I* > 2σ(*I*)
*R*_int_ = 0.020

#### Refinement

*R*[*F* ^2^ > 2σ(*F* ^2^)] = 0.032
*wR*(*F* ^2^) = 0.080
*S* = 1.09
3429 reflections
387 parameters
81 restraints
H-atom parameters constrained
Δρ_max_ = 0.17 e Å^−3^
Δρ_min_ = −0.13 e Å^−3^

### 

Data collection: *CrysAlis CCD* (Oxford Diffraction, 2006 ▶); cell refinement: *CrysAlis RED* (Oxford Diffraction, 2006 ▶); data reduction: *CrysAlis RED*; program(s) used to solve structure: *SHELXS97* (Sheldrick, 2008 ▶); program(s) used to refine structure: *SHELXL97* (Sheldrick, 2008 ▶); molecular graphics: *ORTEP-3* (Farrugia, 1997 ▶) and *Mercury* (Macrae *et al.*, 2008 ▶); software used to prepare material for publication: *SHELXL97*.

## Supplementary Material

Crystal structure: contains datablocks I, global. DOI: 10.1107/S1600536810016636/nk2031sup1.cif

Structure factors: contains datablocks I. DOI: 10.1107/S1600536810016636/nk2031Isup2.hkl

Additional supplementary materials:  crystallographic information; 3D view; checkCIF report

## Figures and Tables

**Table 1:** Hydrogen-bond geometry (Å, °)

*D*—H⋯*A*	*D*—H	H⋯*A*	*D*⋯*A*	*D*—H⋯*A*
N1—H1⋯O1^i^	0.88	1.98	2.831 (2)	163
C13—H13⋯O9^ii^	1.00	2.39	3.292 (3)	149

Symmetry codes: (i) 
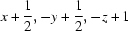
; (ii) 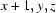.

**Fig. 1.**
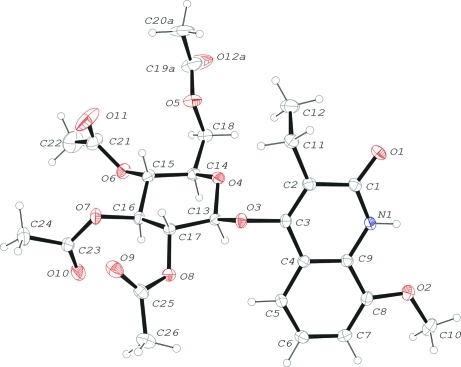
The asymmetric unit with atoms represented as 50 % probability ellipsoids and H atoms shown as small spheres at arbitrary radii. The disorder of acetyl group on O5 has been omitted for clarity.

**Fig. 2.**
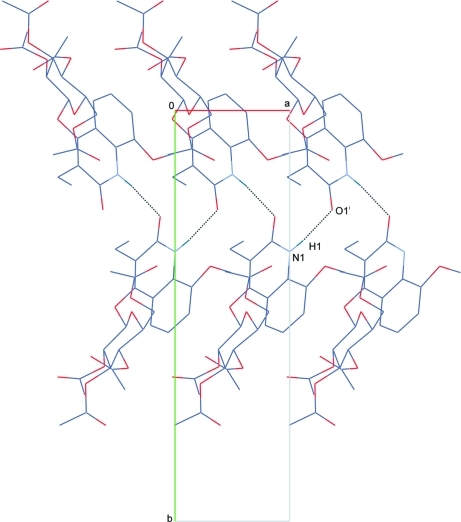
The crystal packing of the title compound showing chains parallel to the a-axis linked via N—H···O H-bonds (dotted lines). The disorder of acetyl group on O5, and all H-atoms (except those which are involved in H-bonding), have been omitted for clarity. Symmetry code: (i) x+0.5, -y+0.5,-z+1.

## supplementary crystallographic information

### Comment

The title compound represents one of the first selectively 4-*O*-glucosylated *N*-unsubstituted 4-hydroxyquinolin-2(1*H*)-ones with potential antimicrobial activity (Kimmel *et al.*, 2010). Several previously prepared saccharide functionalized quinoline derivatives possess interesting bioactivities e.g. as antimalaric agents (Suzuki *et al.*, 2007).

The structure of the title compound (Fig. 1) consists of an essentially planar quinoline ring with the maximum deviation from the best plane being of 0.055 (2)Å for C6 and an oxane ring in classical chair conformation with Cremer & Pople (1975) puckering parameters being Q = 0.586 (2) Å, θ= 11.5 (2) and φ= 309.4 (10)°. The torsion angles describing alignment of peracetylated glucose unit, ethyl in the C2 position and methoxy group in the C8 position C2—C3—O3—C13, C3—O3—C13—O4, C1—C2—C11—C12 and C9—C8—O2—C10 are 103.2 (2), -73.23 (18), 66.1 (3) and -175.86 (19)° respectively. The acetyl group on the O5 was refined using a two-part disorder model with  occupancies being 0.634 (8):0.366 (8). The absolute configurations on C14—C17 correspond to those in starting material and inversion on C13 is in agreement with the well known mechanism of Koenigs-Knorr synthesis. The molecules are linked via N1—H1···O1 H-bonds (Fig. 2, Table 1) into chains parallel to the *a*-axis. The packing of the crystal is stabilized by further C—H···O weak interactions (Table 1).

### Experimental

The title compound was synthesised by Koenigs-Knorr glucosylation of 3-ethyl-4-hydroxy-8-methoxyquinolin-2(1*H*)-one with acetobromo-α-D-glucose in the presence of caesium carbonate in acetonitrile medium as described recently (Kimmel *et al.*, 2010). The crystal used for data collection was obtained by crystallisation from ethanol at room temperature.

### Refinement

The disordered acetyl group was modeled over two sites using similarity restraints to maintain a reasonable geometry and displacement parameters. The two sites are occupied in a 63:37 ratio. Hydrogen atoms were positioned geometrically and refined as riding using standard SHELXTL constraints, with their Uiso set to either 1.2U_eq_ or 1.5U_eq_ (methyl) of their parent atoms. In the absence of significant anomalous scattering, Friedel pairs were merged.

### Crystal data

**Table:**

C_26_H_31_NO_12_	*D*_x_ = 1.298 Mg m^−^^3^
*M**_r_* = 549.52	Melting point = 452–455 K
Orthorhombic, *P*2_1_2_1_2_1_	Mo *K*α radiation, λ = 0.71073 Å
Hall symbol: P 2ac 2ab	Cell parameters from 17385 reflections
*a* = 5.36993 (11) Å	θ = 3.1–27.2°
*b* = 19.2205 (6) Å	µ = 0.10 mm^−^^1^
*c* = 27.2479 (6) Å	*T* = 150 K
*V* = 2812.33 (11) Å^3^	Block, colourless
*Z* = 4	0.40 × 0.40 × 0.30 mm
*F*(000) = 1160	

### Data collection

**Table:**

Kuma KM-4 CCD diffractometer	3429 independent reflections
Radiation source: fine-focus sealed tube	2990 reflections with *I* > 2σ(*I*)
graphite	*R*_int_ = 0.020
Detector resolution: 0.06 pixels mm^-1^	θ_max_ = 27.3°, θ_min_ = 3.1°
ω scan	*h* = −6→6
Absorption correction: multi-scan (*CrysAlis RED*; Oxford Diffraction, 2006)	*k* = −13→24
*T*_min_ = 0.918, *T*_max_ = 0.967	*l* = −34→34
32021 measured reflections	

### Refinement

**Table:**

Refinement on *F*^2^	Primary atom site location: structure-invariant direct methods
Least-squares matrix: full	Secondary atom site location: difference Fourier map
*R*[*F*^2^ > 2σ(*F*^2^)] = 0.032	Hydrogen site location: inferred from neighbouring sites
*wR*(*F*^2^) = 0.080	H-atom parameters constrained
*S* = 1.09	*w* = 1/[σ^2^(*F*_o_^2^) + (0.0384*P*)^2^ + 0.5245*P*] where *P* = (*F*_o_^2^ + 2*F*_c_^2^)/3
3429 reflections	(Δ/σ)_max_ < 0.001
387 parameters	Δρ_max_ = 0.17 e Å^−^^3^
81 restraints	Δρ_min_ = −0.13 e Å^−^^3^

### Special details

**Table:**

Geometry. All esds (except the esd in the dihedral angle between two l.s. planes) are estimated using the full covariance matrix. The cell esds are taken into account individually in the estimation of esds in distances, angles and torsion angles; correlations between esds in cell parameters are only used when they are defined by crystal symmetry. An approximate (isotropic) treatment of cell esds is used for estimating esds involving l.s. planes.
Refinement. Refinement of *F*^2^ against ALL reflections. The weighted *R*-factor wR and goodness of fit *S* are based on *F*^2^, conventional *R*-factors *R* are based on *F*, with *F* set to zero for negative *F*^2^. The threshold expression of *F*^2^ > 2σ(*F*^2^) is used only for calculating *R*-factors(gt) etc. and is not relevant to the choice of reflections for refinement. *R*-factors based on *F*^2^ are statistically about twice as large as those based on *F*, and *R*- factors based on ALL data will be even larger.

### Fractional atomic coordinates and isotropic or equivalent isotropic displacement parameters (Å^2^)

**Table:**

	*x*	*y*	*z*	*U*_iso_*/*U*_eq_	Occ. (<1)
O1	0.8708 (3)	0.25786 (7)	0.53327 (5)	0.0341 (3)	
O2	1.3082 (3)	0.37931 (8)	0.41238 (6)	0.0427 (4)	
O3	0.4748 (3)	0.47871 (7)	0.53992 (5)	0.0271 (3)	
O4	0.6219 (3)	0.48949 (7)	0.61770 (5)	0.0282 (3)	
O5	0.6116 (3)	0.45605 (8)	0.71945 (5)	0.0410 (4)	
O6	0.5932 (3)	0.62765 (8)	0.70831 (5)	0.0350 (3)	
O7	0.2195 (3)	0.66554 (7)	0.64089 (5)	0.0315 (3)	
O8	0.3834 (3)	0.62367 (7)	0.53780 (5)	0.0281 (3)	
O9	−0.0246 (3)	0.64890 (8)	0.54408 (6)	0.0426 (4)	
O10	0.3960 (4)	0.76681 (8)	0.62089 (7)	0.0567 (5)	
O11	0.2632 (5)	0.59852 (14)	0.75466 (8)	0.0878 (9)	
N1	1.0003 (3)	0.34410 (8)	0.48366 (6)	0.0270 (4)	
H1	1.1186	0.3166	0.4726	0.032*	
C1	0.8445 (4)	0.31841 (10)	0.51878 (7)	0.0275 (4)	
C2	0.6558 (4)	0.36595 (10)	0.53815 (7)	0.0277 (4)	
C3	0.6499 (4)	0.43204 (10)	0.52114 (7)	0.0252 (4)	
C4	0.8076 (4)	0.45683 (10)	0.48212 (7)	0.0248 (4)	
C5	0.7871 (4)	0.52319 (10)	0.46025 (7)	0.0306 (4)	
H5	0.6672	0.5556	0.4719	0.037*	
C6	0.9409 (5)	0.54054 (11)	0.42219 (8)	0.0369 (5)	
H6	0.9236	0.5848	0.4071	0.044*	
C7	1.1230 (5)	0.49464 (11)	0.40506 (8)	0.0363 (5)	
H7	1.2315	0.5083	0.3793	0.044*	
C8	1.1447 (4)	0.42976 (11)	0.42563 (7)	0.0312 (5)	
C9	0.9848 (4)	0.41033 (10)	0.46433 (7)	0.0256 (4)	
C10	1.4892 (5)	0.39485 (14)	0.37598 (8)	0.0436 (6)	
H10A	1.6029	0.3553	0.3724	0.065*	
H10B	1.5837	0.4362	0.3858	0.065*	
H10C	1.4058	0.4038	0.3446	0.065*	
C11	0.4782 (5)	0.33763 (11)	0.57576 (8)	0.0391 (5)	
H11A	0.3952	0.2960	0.5620	0.047*	
H11B	0.3480	0.3729	0.5821	0.047*	
C12	0.5982 (7)	0.31787 (14)	0.62423 (9)	0.0614 (8)	
H12A	0.4688	0.3042	0.6477	0.092*	
H12B	0.6905	0.3578	0.6372	0.092*	
H12C	0.7128	0.2789	0.6191	0.092*	
C13	0.5741 (4)	0.52634 (10)	0.57359 (6)	0.0238 (4)	
H13	0.7309	0.5474	0.5605	0.029*	
C14	0.7213 (4)	0.53372 (11)	0.65513 (7)	0.0275 (4)	
H14	0.8610	0.5618	0.6411	0.033*	
C15	0.5099 (4)	0.58234 (11)	0.66957 (7)	0.0269 (4)	
H15	0.3631	0.5546	0.6809	0.032*	
C16	0.4377 (4)	0.62670 (10)	0.62631 (7)	0.0255 (4)	
H16	0.5763	0.6595	0.6183	0.031*	
C17	0.3777 (4)	0.58191 (10)	0.58157 (6)	0.0240 (4)	
H17	0.2101	0.5599	0.5855	0.029*	
C18	0.8190 (4)	0.48969 (12)	0.69638 (8)	0.0348 (5)	
H18A	0.9367	0.4545	0.6835	0.042*	
H18B	0.9082	0.5191	0.7205	0.042*	
C21	0.4515 (6)	0.63038 (14)	0.74961 (9)	0.0497 (7)	
C22	0.5634 (8)	0.68002 (18)	0.78556 (10)	0.0779 (11)	
H22A	0.5080	0.6680	0.8188	0.117*	
H22B	0.5103	0.7275	0.7777	0.117*	
H22C	0.7454	0.6771	0.7838	0.117*	
C23	0.2251 (4)	0.73549 (11)	0.63784 (8)	0.0308 (5)	
C24	−0.0071 (5)	0.76662 (13)	0.65782 (10)	0.0443 (6)	
H24A	0.0278	0.8137	0.6698	0.067*	
H24B	−0.0689	0.7379	0.6849	0.067*	
H24C	−0.1333	0.7688	0.6319	0.067*	
C25	0.1693 (4)	0.65329 (11)	0.52218 (8)	0.0322 (5)	
C26	0.2118 (5)	0.69197 (13)	0.47544 (9)	0.0469 (6)	
H26A	0.0592	0.6913	0.4557	0.070*	
H26B	0.3469	0.6698	0.4570	0.070*	
H26C	0.2573	0.7402	0.4829	0.070*	
C19B	0.647 (2)	0.4064 (9)	0.7485 (5)	0.069 (3)	0.366 (8)
C20B	0.435 (3)	0.3921 (15)	0.7846 (9)	0.060 (4)	0.366 (8)
H20D	0.5040	0.3813	0.8170	0.090*	0.366 (8)
H20E	0.3359	0.3526	0.7729	0.090*	0.366 (8)
H20F	0.3280	0.4334	0.7870	0.090*	0.366 (8)
O12B	0.8449 (12)	0.3784 (5)	0.7519 (4)	0.093 (3)	0.366 (8)
C19A	0.6517 (12)	0.4270 (4)	0.7636 (3)	0.0563 (18)	0.634 (8)
C20A	0.415 (2)	0.3870 (9)	0.7769 (5)	0.072 (3)	0.634 (8)
H20A	0.4502	0.3554	0.8043	0.108*	0.634 (8)
H20B	0.3589	0.3601	0.7485	0.108*	0.634 (8)
H20C	0.2846	0.4199	0.7866	0.108*	0.634 (8)
O12A	0.8390 (7)	0.4347 (4)	0.78546 (18)	0.106 (2)	0.634 (8)

### Atomic displacement parameters (Å^2^)

**Table:**

	*U*^11^	*U*^22^	*U*^33^	*U*^12^	*U*^13^	*U*^23^
O1	0.0389 (8)	0.0191 (7)	0.0443 (8)	−0.0035 (7)	−0.0025 (8)	0.0028 (6)
O2	0.0448 (9)	0.0399 (9)	0.0435 (9)	0.0117 (8)	0.0165 (8)	0.0028 (7)
O3	0.0281 (7)	0.0235 (7)	0.0297 (6)	−0.0004 (6)	0.0014 (6)	−0.0070 (6)
O4	0.0362 (8)	0.0239 (7)	0.0245 (6)	−0.0009 (7)	0.0008 (6)	0.0002 (5)
O5	0.0425 (9)	0.0482 (10)	0.0324 (7)	−0.0075 (9)	−0.0021 (8)	0.0118 (7)
O6	0.0384 (8)	0.0382 (8)	0.0284 (7)	−0.0048 (8)	−0.0019 (7)	−0.0103 (6)
O7	0.0265 (7)	0.0268 (7)	0.0411 (8)	0.0006 (7)	0.0070 (7)	−0.0093 (7)
O8	0.0290 (7)	0.0282 (7)	0.0270 (6)	0.0002 (6)	0.0013 (6)	0.0025 (6)
O9	0.0273 (8)	0.0374 (9)	0.0632 (11)	0.0003 (7)	−0.0016 (8)	0.0107 (8)
O10	0.0527 (11)	0.0316 (8)	0.0859 (13)	0.0056 (9)	0.0298 (11)	0.0093 (9)
O11	0.0876 (17)	0.125 (2)	0.0504 (12)	−0.0470 (17)	0.0358 (12)	−0.0397 (13)
N1	0.0300 (8)	0.0204 (8)	0.0307 (8)	0.0027 (8)	0.0022 (8)	−0.0023 (7)
C1	0.0309 (10)	0.0215 (10)	0.0301 (10)	−0.0050 (9)	−0.0028 (9)	−0.0030 (8)
C2	0.0327 (10)	0.0243 (10)	0.0261 (9)	−0.0053 (9)	−0.0006 (9)	−0.0022 (8)
C3	0.0267 (10)	0.0232 (10)	0.0257 (9)	0.0001 (9)	−0.0009 (8)	−0.0046 (8)
C4	0.0306 (10)	0.0228 (10)	0.0211 (8)	−0.0002 (9)	−0.0019 (8)	−0.0012 (8)
C5	0.0405 (11)	0.0232 (10)	0.0280 (9)	0.0066 (10)	0.0011 (10)	−0.0018 (8)
C6	0.0526 (14)	0.0266 (10)	0.0314 (10)	0.0016 (11)	0.0022 (11)	0.0046 (9)
C7	0.0441 (13)	0.0352 (12)	0.0295 (10)	−0.0011 (11)	0.0088 (10)	0.0040 (9)
C8	0.0348 (11)	0.0297 (11)	0.0291 (10)	0.0020 (10)	0.0035 (10)	−0.0041 (9)
C9	0.0301 (10)	0.0220 (9)	0.0247 (9)	−0.0002 (9)	−0.0013 (9)	−0.0013 (8)
C10	0.0365 (12)	0.0600 (16)	0.0343 (11)	0.0046 (12)	0.0084 (10)	−0.0072 (11)
C11	0.0511 (14)	0.0244 (10)	0.0417 (12)	−0.0068 (11)	0.0122 (12)	−0.0019 (9)
C12	0.105 (2)	0.0386 (14)	0.0412 (13)	−0.0053 (17)	0.0132 (17)	0.0093 (11)
C13	0.0262 (9)	0.0231 (10)	0.0221 (8)	−0.0013 (9)	0.0018 (8)	−0.0009 (8)
C14	0.0259 (10)	0.0302 (11)	0.0264 (9)	−0.0040 (9)	0.0020 (8)	0.0004 (8)
C15	0.0296 (10)	0.0276 (10)	0.0236 (9)	−0.0070 (10)	0.0016 (8)	−0.0045 (8)
C16	0.0223 (9)	0.0247 (10)	0.0296 (10)	0.0000 (8)	0.0044 (8)	−0.0034 (8)
C17	0.0236 (9)	0.0228 (9)	0.0256 (9)	−0.0019 (9)	0.0028 (8)	0.0009 (8)
C18	0.0314 (11)	0.0381 (13)	0.0351 (11)	−0.0016 (11)	−0.0024 (9)	0.0043 (10)
C21	0.0624 (18)	0.0572 (16)	0.0293 (11)	−0.0063 (15)	0.0029 (12)	−0.0125 (11)
C22	0.106 (3)	0.086 (2)	0.0415 (14)	−0.011 (2)	−0.0116 (18)	−0.0300 (15)
C23	0.0347 (11)	0.0293 (11)	0.0285 (10)	0.0024 (10)	0.0012 (9)	−0.0034 (9)
C24	0.0385 (12)	0.0371 (12)	0.0574 (15)	0.0064 (12)	0.0064 (12)	−0.0096 (11)
C25	0.0342 (12)	0.0221 (10)	0.0403 (11)	−0.0028 (9)	−0.0071 (10)	0.0008 (9)
C26	0.0532 (15)	0.0409 (13)	0.0467 (13)	−0.0018 (13)	−0.0088 (12)	0.0115 (11)
C19B	0.046 (4)	0.102 (7)	0.060 (6)	0.000 (4)	−0.008 (4)	0.047 (5)
C20B	0.047 (5)	0.080 (8)	0.054 (6)	−0.003 (6)	−0.009 (4)	0.009 (6)
O12B	0.048 (3)	0.129 (6)	0.102 (6)	0.011 (4)	0.000 (4)	0.088 (5)
C19A	0.036 (2)	0.089 (5)	0.045 (3)	0.007 (3)	0.004 (2)	0.029 (3)
C20A	0.055 (4)	0.094 (6)	0.066 (6)	−0.010 (4)	0.010 (4)	0.054 (5)
O12A	0.050 (2)	0.195 (6)	0.072 (3)	−0.024 (3)	−0.017 (2)	0.077 (4)

### Geometric parameters (Å, °)

**Table:**

O1—C1	1.237 (2)	C11—H11A	0.9900
O2—C8	1.357 (3)	C11—H11B	0.9900
O2—C10	1.421 (3)	C12—H12A	0.9800
O3—C3	1.397 (2)	C12—H12B	0.9800
O3—C13	1.402 (2)	C12—H12C	0.9800
O4—C13	1.419 (2)	C13—C17	1.516 (3)
O4—C14	1.431 (2)	C13—H13	1.0000
O5—C19B	1.256 (15)	C14—C18	1.502 (3)
O5—C19A	1.342 (8)	C14—C15	1.522 (3)
O5—C18	1.433 (3)	C14—H14	1.0000
O6—C21	1.359 (3)	C15—C16	1.506 (3)
O6—C15	1.440 (2)	C15—H15	1.0000
O7—C23	1.347 (3)	C16—C17	1.527 (3)
O7—C16	1.445 (2)	C16—H16	1.0000
O8—C25	1.351 (3)	C17—H17	1.0000
O8—C17	1.438 (2)	C18—H18A	0.9900
O9—C25	1.203 (3)	C18—H18B	0.9900
O10—C23	1.191 (3)	C21—C22	1.494 (4)
O11—C21	1.190 (3)	C22—H22A	0.9800
N1—C1	1.364 (3)	C22—H22B	0.9800
N1—C9	1.380 (3)	C22—H22C	0.9800
N1—H1	0.8800	C23—C24	1.486 (3)
C1—C2	1.463 (3)	C24—H24A	0.9800
C2—C3	1.353 (3)	C24—H24B	0.9800
C2—C11	1.502 (3)	C24—H24C	0.9800
C3—C4	1.440 (3)	C25—C26	1.492 (3)
C4—C9	1.393 (3)	C26—H26A	0.9800
C4—C5	1.412 (3)	C26—H26B	0.9800
C5—C6	1.367 (3)	C26—H26C	0.9800
C5—H5	0.9500	C19B—O12B	1.195 (12)
C6—C7	1.397 (3)	C19B—C20B	1.529 (14)
C6—H6	0.9500	C20B—H20D	0.9800
C7—C8	1.372 (3)	C20B—H20E	0.9800
C7—H7	0.9500	C20B—H20F	0.9800
C8—C9	1.410 (3)	C19A—O12A	1.179 (7)
C10—H10A	0.9800	C19A—C20A	1.529 (10)
C10—H10B	0.9800	C20A—H20A	0.9800
C10—H10C	0.9800	C20A—H20B	0.9800
C11—C12	1.518 (4)	C20A—H20C	0.9800
			
C8—O2—C10	118.57 (17)	O4—C14—H14	109.1
C3—O3—C13	113.78 (15)	C18—C14—H14	109.1
C13—O4—C14	112.00 (14)	C15—C14—H14	109.1
C19B—O5—C19A	25.1 (8)	O6—C15—C16	108.14 (16)
C19B—O5—C18	120.2 (6)	O6—C15—C14	109.22 (17)
C19A—O5—C18	117.1 (3)	C16—C15—C14	109.70 (15)
C21—O6—C15	117.16 (18)	O6—C15—H15	109.9
C23—O7—C16	118.72 (17)	C16—C15—H15	109.9
C25—O8—C17	118.58 (16)	C14—C15—H15	109.9
C1—N1—C9	124.38 (18)	O7—C16—C15	106.62 (15)
C1—N1—H1	117.8	O7—C16—C17	109.87 (15)
C9—N1—H1	117.8	C15—C16—C17	111.11 (16)
O1—C1—N1	119.63 (19)	O7—C16—H16	109.7
O1—C1—C2	123.48 (19)	C15—C16—H16	109.7
N1—C1—C2	116.87 (17)	C17—C16—H16	109.7
C3—C2—C1	118.65 (18)	O8—C17—C13	105.04 (14)
C3—C2—C11	123.99 (19)	O8—C17—C16	110.07 (15)
C1—C2—C11	117.36 (17)	C13—C17—C16	111.41 (16)
C2—C3—O3	119.58 (17)	O8—C17—H17	110.1
C2—C3—C4	123.35 (18)	C13—C17—H17	110.1
O3—C3—C4	116.99 (16)	C16—C17—H17	110.1
C9—C4—C5	119.09 (18)	O5—C18—C14	108.13 (17)
C9—C4—C3	116.51 (17)	O5—C18—H18A	110.1
C5—C4—C3	124.36 (19)	C14—C18—H18A	110.1
C6—C5—C4	119.6 (2)	O5—C18—H18B	110.1
C6—C5—H5	120.2	C14—C18—H18B	110.1
C4—C5—H5	120.2	H18A—C18—H18B	108.4
C5—C6—C7	121.5 (2)	O11—C21—O6	123.5 (2)
C5—C6—H6	119.3	O11—C21—C22	126.5 (3)
C7—C6—H6	119.3	O6—C21—C22	110.0 (3)
C8—C7—C6	119.8 (2)	C21—C22—H22A	109.5
C8—C7—H7	120.1	C21—C22—H22B	109.5
C6—C7—H7	120.1	H22A—C22—H22B	109.5
O2—C8—C7	126.5 (2)	C21—C22—H22C	109.5
O2—C8—C9	113.80 (18)	H22A—C22—H22C	109.5
C7—C8—C9	119.6 (2)	H22B—C22—H22C	109.5
N1—C9—C4	120.02 (18)	O10—C23—O7	123.1 (2)
N1—C9—C8	119.53 (18)	O10—C23—C24	125.8 (2)
C4—C9—C8	120.42 (18)	O7—C23—C24	111.1 (2)
O2—C10—H10A	109.5	C23—C24—H24A	109.5
O2—C10—H10B	109.5	C23—C24—H24B	109.5
H10A—C10—H10B	109.5	H24A—C24—H24B	109.5
O2—C10—H10C	109.5	C23—C24—H24C	109.5
H10A—C10—H10C	109.5	H24A—C24—H24C	109.5
H10B—C10—H10C	109.5	H24B—C24—H24C	109.5
C2—C11—C12	114.5 (2)	O9—C25—O8	123.39 (19)
C2—C11—H11A	108.6	O9—C25—C26	126.2 (2)
C12—C11—H11A	108.6	O8—C25—C26	110.40 (19)
C2—C11—H11B	108.6	C25—C26—H26A	109.5
C12—C11—H11B	108.6	C25—C26—H26B	109.5
H11A—C11—H11B	107.6	H26A—C26—H26B	109.5
C11—C12—H12A	109.5	C25—C26—H26C	109.5
C11—C12—H12B	109.5	H26A—C26—H26C	109.5
H12A—C12—H12B	109.5	H26B—C26—H26C	109.5
C11—C12—H12C	109.5	O12B—C19B—O5	121.7 (11)
H12A—C12—H12C	109.5	O12B—C19B—C20B	122.2 (15)
H12B—C12—H12C	109.5	O5—C19B—C20B	115.4 (14)
O3—C13—O4	107.29 (15)	O12A—C19A—O5	122.6 (6)
O3—C13—C17	106.83 (15)	O12A—C19A—C20A	130.6 (9)
O4—C13—C17	110.86 (14)	O5—C19A—C20A	106.8 (7)
O3—C13—H13	110.6	C19A—C20A—H20A	109.5
O4—C13—H13	110.6	C19A—C20A—H20B	109.5
C17—C13—H13	110.6	H20A—C20A—H20B	109.5
O4—C14—C18	109.20 (17)	C19A—C20A—H20C	109.5
O4—C14—C15	105.71 (16)	H20A—C20A—H20C	109.5
C18—C14—C15	114.39 (16)	H20B—C20A—H20C	109.5
			
C9—N1—C1—O1	178.86 (18)	C13—O4—C14—C15	68.72 (19)
C9—N1—C1—C2	−2.5 (3)	C21—O6—C15—C16	113.7 (2)
O1—C1—C2—C3	177.27 (19)	C21—O6—C15—C14	−126.9 (2)
N1—C1—C2—C3	−1.3 (3)	O4—C14—C15—O6	178.44 (14)
O1—C1—C2—C11	−3.2 (3)	C18—C14—C15—O6	58.3 (2)
N1—C1—C2—C11	178.23 (18)	O4—C14—C15—C16	−63.19 (19)
C1—C2—C3—O3	−178.62 (16)	C18—C14—C15—C16	176.64 (18)
C11—C2—C3—O3	1.9 (3)	C23—O7—C16—C15	124.02 (19)
C1—C2—C3—C4	4.9 (3)	C23—O7—C16—C17	−115.48 (18)
C11—C2—C3—C4	−174.59 (19)	O6—C15—C16—O7	−67.3 (2)
C13—O3—C3—C2	103.2 (2)	C14—C15—C16—O7	173.66 (15)
C13—O3—C3—C4	−80.1 (2)	O6—C15—C16—C17	173.00 (16)
C2—C3—C4—C9	−4.7 (3)	C14—C15—C16—C17	54.0 (2)
O3—C3—C4—C9	178.79 (16)	C25—O8—C17—C13	147.94 (17)
C2—C3—C4—C5	173.0 (2)	C25—O8—C17—C16	−92.0 (2)
O3—C3—C4—C5	−3.5 (3)	O3—C13—C17—O8	−74.60 (17)
C9—C4—C5—C6	0.0 (3)	O4—C13—C17—O8	168.81 (14)
C3—C4—C5—C6	−177.7 (2)	O3—C13—C17—C16	166.26 (15)
C4—C5—C6—C7	−1.6 (3)	O4—C13—C17—C16	49.7 (2)
C5—C6—C7—C8	2.0 (3)	O7—C16—C17—O8	79.57 (19)
C10—O2—C8—C7	4.6 (3)	C15—C16—C17—O8	−162.69 (16)
C10—O2—C8—C9	−175.86 (18)	O7—C16—C17—C13	−164.32 (15)
C6—C7—C8—O2	178.7 (2)	C15—C16—C17—C13	−46.6 (2)
C6—C7—C8—C9	−0.8 (3)	C19B—O5—C18—C14	166.2 (9)
C1—N1—C9—C4	2.8 (3)	C19A—O5—C18—C14	−165.4 (4)
C1—N1—C9—C8	−175.50 (19)	O4—C14—C18—O5	−67.2 (2)
C5—C4—C9—N1	−177.02 (18)	C15—C14—C18—O5	51.0 (2)
C3—C4—C9—N1	0.8 (3)	C15—O6—C21—O11	−1.4 (4)
C5—C4—C9—C8	1.2 (3)	C15—O6—C21—C22	−179.6 (2)
C3—C4—C9—C8	179.01 (18)	C16—O7—C23—O10	4.7 (3)
O2—C8—C9—N1	−2.1 (3)	C16—O7—C23—C24	−175.71 (17)
C7—C8—C9—N1	177.45 (19)	C17—O8—C25—O9	3.1 (3)
O2—C8—C9—C4	179.66 (18)	C17—O8—C25—C26	−177.82 (17)
C7—C8—C9—C4	−0.8 (3)	C19A—O5—C19B—O12B	−102 (3)
C3—C2—C11—C12	−114.4 (2)	C18—O5—C19B—O12B	−12.3 (19)
C1—C2—C11—C12	66.1 (3)	C19A—O5—C19B—C20B	68 (2)
C3—O3—C13—O4	−73.23 (18)	C18—O5—C19B—C20B	158.4 (14)
C3—O3—C13—C17	167.84 (14)	C19B—O5—C19A—O12A	112 (2)
C14—O4—C13—O3	−179.20 (15)	C18—O5—C19A—O12A	8.2 (9)
C14—O4—C13—C17	−62.9 (2)	C19B—O5—C19A—C20A	−69 (2)
C13—O4—C14—C18	−167.76 (16)	C18—O5—C19A—C20A	−172.8 (8)

### Hydrogen-bond geometry (Å, °)

**Table:**

*D*—H···*A*	*D*—H	H···*A*	*D*···*A*	*D*—H···*A*
N1—H1···O1^i^	0.88	1.98	2.831 (2)	163
C13—H13···O9^ii^	1.00	2.39	3.292 (3)	149

Symmetry codes: (i) *x*+1/2, −*y*+1/2, −*z*+1; (ii) *x*+1, *y*, *z*.
